# Malignancy in Pediatric Hyperfunctioning Thyroid Nodules: A Case Report and Literature Review

**DOI:** 10.1111/cen.70165

**Published:** 2026-05-15

**Authors:** Cristina Clausi, Simona Censi, Jacopo Maria Arnone, Giulia Messina, Ilaria Piva, Fausto Cortese, Loris Bertazza, Susi Barollo, Francesca Galuppini, Marco Zarantonello, Maurizio Iacobone, Gianmaria Pennelli, Caterina Mian

**Affiliations:** ^1^ Department of Medicine (DIMED), Endocrinology Unit University of Padua Padua Italy; ^2^ Endocrine Unit University Hospital of Padua Padua Italy; ^3^ Endocrinology Unit University Hospital of Padua Padua Italy; ^4^ Familial Cancer Unit Veneto Institute of Oncology (IOV)‐IRCSS Padua Italy; ^5^ Surgical Pathology & Cytopathology Unit University Hospital of Padua Padua Italy; ^6^ Department of Surgery, Endocrine Surgery Unit, Oncology and Gastroenterology (DiSCOG) University of Padua Padua Italy

**Keywords:** carcinoma, child, follicular, GNAS proteins, hyperthyroidism, mutation, thyroid neoplasms, thyroid nodule

## Abstract

**Background:**

Thyroid nodules are rare in children but carry a markedly higher risk of malignancy compared to adults (20%–26% vs. 5%). Hyperfunctioning thyroid nodules are exceptionally uncommon in the pediatric population and are typically benign. We describe a rare case of a hyperfunctioning thyroid nodule in a prepubertal child that was ultimately diagnosed as an angioinvasive encapsulated follicular carcinoma, arising in the context of the WHO entity ‘follicular adenoma showing papillary architecture’. In addition, we provide a comprehensive review of published pediatric cases of hyperfunctioning thyroid nodules with malignant histology.

**Case Presentation:**

A 12‐year‐old boy presented with a left‐sided thyroid nodule detected on ultrasound after cervical swelling. Laboratory evaluation revealed suppressed thyroid‐stimulating hormone (TSH), with free thyroxine (fT4) and free triiodothyronine (fT3) within the reference range. Serial ultrasound examinations revealed a progressively enlarging mildly hypoechoic nodule, autonomously functioning on thyroid scintigraphy. Fine‐needle aspiration cytology (FNAC) was classified as TIR2. Surgical excision showed a follicular‐patterned neoplasm showing papillary features, focal capsular and vascular invasion and no lymph node metastases. Comprehensive molecular analysis identified a pathogenic somatic variant in the *GNAS* gene, whereas no alterations were detected in *TSHR*, *BRAF*, *RAS* genes, the *TERT* promoter, or *DICER1*.

**Conclusion:**

This case highlights the importance of an integrated evaluation in pediatric thyroid nodules, particularly when hyperfunction coexists with indeterminate or suspicious imaging features. Follicular‐patterned thyroid neoplasms showing papillary architecture pose diagnostic challenges in children, and molecular analysis may assist risk stratification and elucidate pathogenetic mechanisms linking autonomous function and malignant transformation.

## 1 Introduction

Thyroid nodules are uncommon in children, with an estimated prevalence of 1%–2%, but they carry a significantly higher risk of malignancy compared with adults, 20%–26% versus approximately 5% [1, 2, 3]. Pediatric nodules differ in biological behaviour, histology and molecular profile from their adult counterparts, thus requiring an individualised approach. Autonomously functioning thyroid nodules (AFTNs), also known as ‘hot’ nodules, have traditionally been considered benign, largely based on adult data. Histologically, these nodules are typically represented by follicular adenomas exhibiting features of autonomous hormone production, commonly referred to as Plummer's adenoma, a benign hyperfunctioning thyroid adenoma. However, the recent WHO classification of thyroid tumours recognises ‘follicular thyroid adenoma showing papillary architecture’ as a benign, non‐invasive follicular cell‐derived neoplasm characterised by papillary architecture in the absence of the nuclear features of papillary thyroid carcinoma (PTC) and is often associated with autonomous hyperfunction [4]. The existence and definition of a malignant counterpart remain uncertain. The assumption of an underlying benignity has led to the long‐standing recommendation to avoid fine‐needle aspiration biopsy (FNAB) in hot nodules [1, 2]. Nevertheless, emerging pediatric evidence challenges this assumption. Although rare, malignancy arising in AFTNs has been documented, including follicular thyroid carcinoma (FTC) and the follicular variant of papillary thyroid carcinoma (FVPTC). However, the malignant evolution of a follicular thyroid adenoma showing papillary architecture mentioned above has not been documented to date. These follicular‐patterned tumours are particularly challenging to diagnose cytologically, as capsular and vascular invasion, the defining features of malignancy, cannot be assessed by FNAB. Furthermore, activating mutations in *GNAS* (G protein subunit alpha stimulating) or *TSHR* (Thyroid‐Stimulating Hormone Receptor), which are rarely identified in adult thyroid carcinomas, have been reported in malignant pediatric AFTNs [5, 6, 7], suggesting a potential mechanistic link between hyperfunction and tumorigenesis. Given the rarity of pediatric AFTNs and the possibility of underrecognized malignancy, careful evaluation is warranted. Here, we describe a previously unreported case of an encapsulated angioinvasive follicular carcinoma with extensive papillary architecture arising in a hyperfunctioning thyroid nodule in a prepubertal boy. The diagnosis is supported by the presence of capsular and vascular invasion, consistent with follicular carcinoma. Although papillary structures are present, the absence of the typical nuclear features excludes a diagnosis of PTC. We also provide a structured narrative review of the literature to clarify the prevalence, histologic spectrum and molecular features of malignancy in pediatric AFTNs, and to inform clinical decision‐making in this complex scenario.

## 2 Case Presentation (Patient's Case)

**Figure 1:**
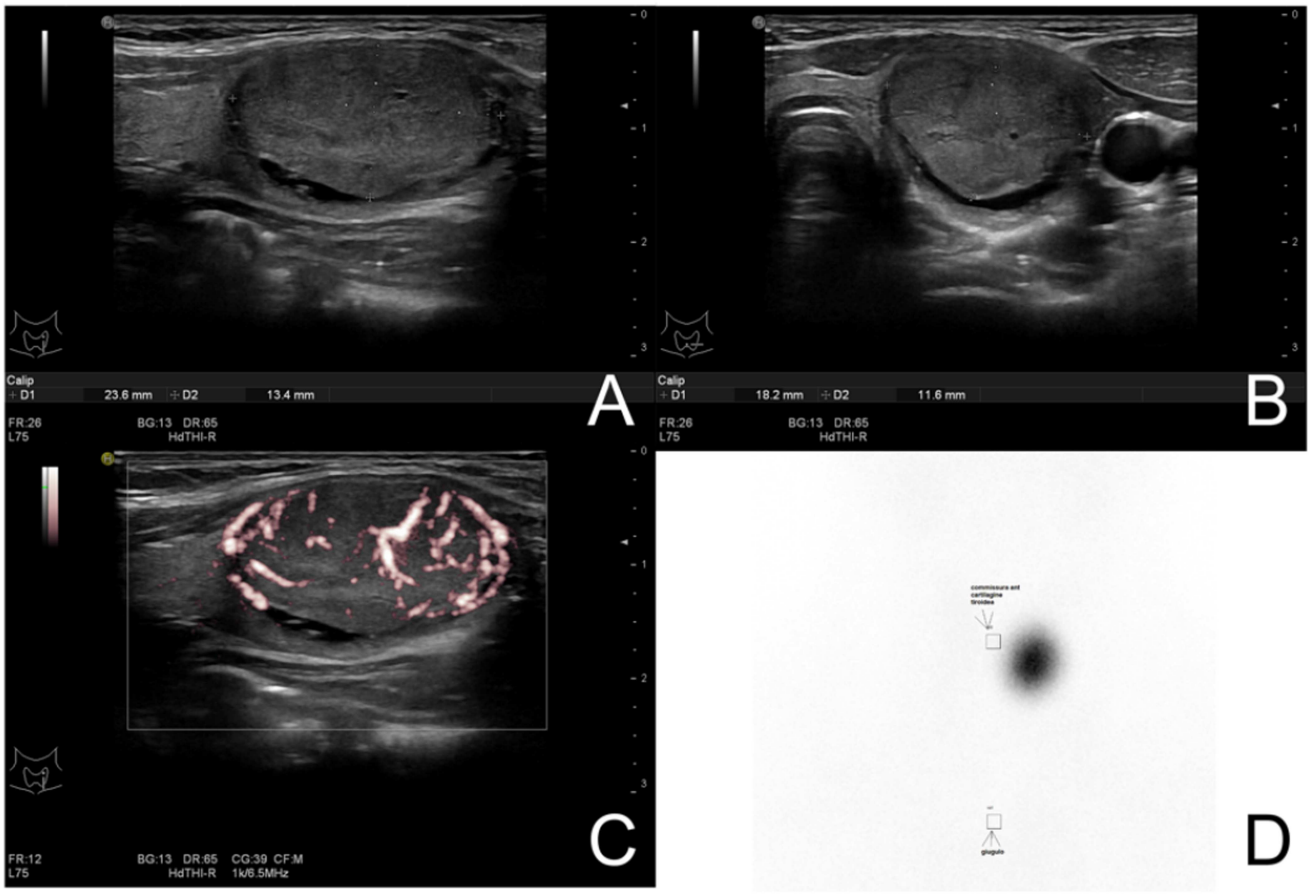
Multimodal imaging of the left thyroid lobe. Neck ultrasounds of the left thyroid lobe showing an ovoid (24 × 13 × 18 mm), mildly hypoechoic solid nodule, located in the upper pole of the left thyroid lobe, with smooth margins and a peripheral vascular flow. (A) Longitudinal view; (B) transverse view; (C) longitudinal view with mixed but predominantly peripheral flow on Colour Doppler. (D) ⁹⁹ᵐTc‐pertechnetate thyroid scintigraphy showing intense focal uptake in the upper pole of the left thyroid lobe with suppression of the surrounding thyroid tissue, consistent with an autonomously functioning thyroid nodule. These findings illustrate that autonomous function may coexist with non‐specific ultrasonographic features, and therefore does not reliably exclude malignancy in pediatric patients.

**Figure 2:**
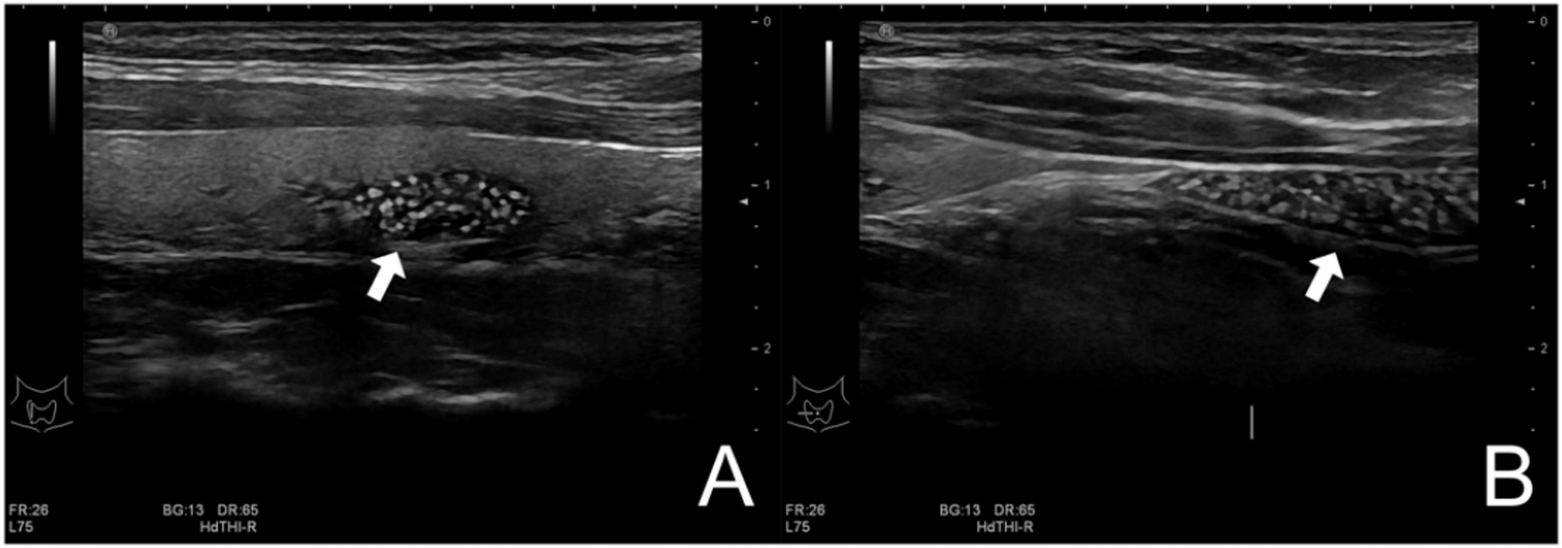
Ultrasound of the right thyroid lobe showing a 10 × 4 mm hypoechoic area with multiple fine hyperechoic foci (*starry‐sky* appearance), consistent with intrathyroidal thymic tissue; (A) comparative view of the thymus versus adjacent thyroid parenchyma; (B) longitudinal view of the intrathyroidal thymic tissue. This finding is relevant in the differential diagnosis, as intrathyroidal thymic tissue may mimic suspicious thyroid lesions on ultrasound in children.

**Figure 3:**
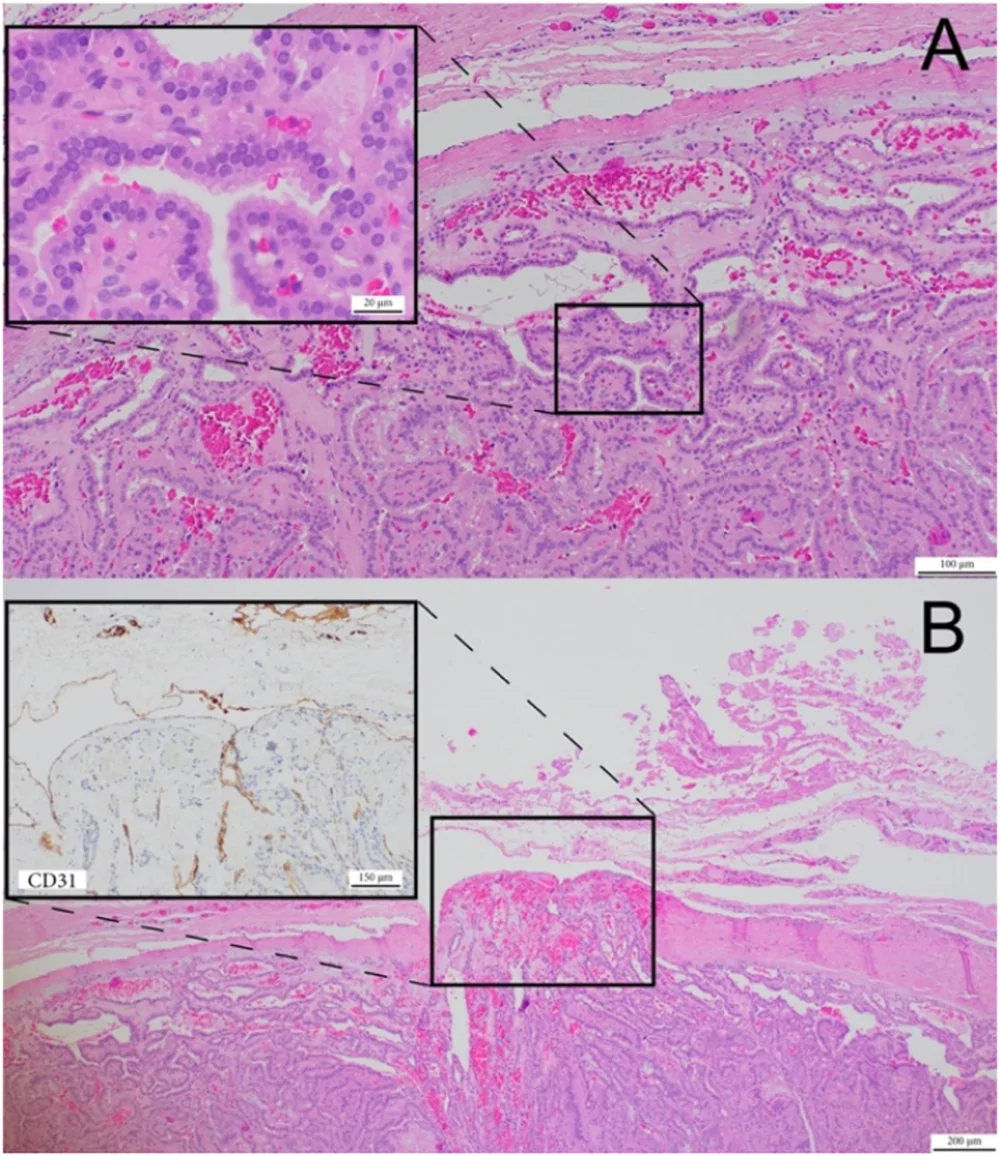
Histopathology features of the neoplasia. Encapsulated follicular lesion showing papillary architecture and histological features of hyperfunction (10x EE). (A) Higher magnification (upper left inset, 40×, H&E) showing nuclei lacking the typical features of papillary thyroid carcinoma (score 0 according to Nikiforov et al. [9]); (B) focal capsular infiltration and vascular invasion, despite a predominantly expansive growth pattern (H&E, 4×). The inset (upper left, CD31 immunohistochemistry, 10×) highlights endothelial cells lining small and large vessels, confirming vascular invasion by demonstrating endothelium adherent to neoplastic cells. Capsular infiltration and vascular invasion are fundamental diagnostic criteria to diagnose the neoplasia as malignant, considering the absence of papillary‐like nuclear atypia. Furthermore, haematic vascular invasion is the histological factor that most strongly affects patient prognosis [11].

A 12‐year‐old boy was referred to the Endocrinology Unit of the University Hospital of Padua in early 2024 for evaluation of a left‐sided cervical swelling. He reported no compressive symptoms or systemic signs of thyroid dysfunction. Physical examination revealed a firm, mobile, painless nodule in the upper pole of the left thyroid lobe. There was no history of neck irradiation, familial thyroid disease, or hereditary syndromes of relevance. His past medical history was notable only for asthma, managed with inhaled budesonide/formoterol. Neck ultrasound (US) revealed an ovoid, mildly hypoechoic solid nodule, located in the upper pole of the left thyroid lobe, with smooth margins and a peripheral thin halo, and predominantly perinodular and slight intranodular flow, measuring 19 × 15 × 13 mm in March 2024, which increased to 24 × 13 × 18 mm by November 2024 (Figure 1A–C). The remaining thyroid tissue appeared homogeneous, and no suspicious lymph nodes were identified. A small hypoechoic area with a *starry‐sky* pattern in the contralateral lobe was consistent with possible ectopic intrathyroidal thymic tissue (Figure 2). Laboratory testing, measured using commercially available immunoassays (Roche, Rotkreuz, Switzerland), showed low TSH (0.22 mIU/L, reference range: 0.6–4.7 mIU/L), with normal fT4 (17 pmol/L, reference range: 9–22 pmol/L) and fT3 in the high half of normal range (6.4 pmol/L, reference range: 3.9–6.8 pmol/L). Serum calcitonin (CT) was within the normal range (6.1 ng/L, reference range: < 10 ng/L). Serum thyroglobulin (Tg) was 24.6 µg/L (reference range: 1.6–50.0 µg/L). Anti‐thyroglobulin antibodies (TgAb) were slightly elevated, with a peak value of 6 IU/mL (reference range: < 4.5 IU/mL), while anti‐thyroid peroxidase antibodies (TPOAb) were negative (< 34 IU/mL). FNAC of the left nodule, performed in April 2024 at a peripheral centre, resulted in TIR‐2 according to the Italian consensus for the classification and reporting of thyroid cytology (SIAPEC‐IAP 2014) [8]. Thyroid scintigraphy with technetium‐99m (⁹⁹ᵐTc) pertechnetate, performed in November 2024, revealed an intense focal uptake in the upper pole of the left thyroid lobe, corresponding to the palpable nodule, with a suppression of the surrounding thyroid parenchyma, consistent with an AFTN (Figure 1D). Due to the progressive growth observed on US and the subclinical hyperthyroidism, a left hemithyroidectomy was performed in March 2025. Histopathological examination, performed by two expert pathologists (F.G. and G.P.), revealed a 2.4‐cm encapsulated thyroid lesion with extensive papillary architecture (> 50% of the examined lesion surface), without nuclear atypia (nuclear score 0, according to the nuclear scoring system proposed by Nikiforov et al. [9], with focal findings of infiltration of the tumour capsule associated with haematic vascular invasion, diagnosed as ‘encapsulated angio‐invasive follicular carcinoma showing papillary architecture’ arising from a follicular adenoma with papillary architecture. Necrosis was absent, and the mitotic index was 1–2 per 2 mm^2^. Ki67 proliferation index was about 5%. There was no evidence of infiltration of the thyroid capsule or perithyroidal soft tissues and four regional lymph nodes (ranging from 0.1 to 0.3 cm) were assessed without evidence of metastasis. The carcinoma was staged as pT1a N0, according to the UICC 8th edition [10]. Immunohistochemistry for *BRAF V600E* was negative (Figure 3).

A detailed differential diagnostic assessment was performed to distinguish this lesion from other follicular cell‐derived low‐risk neoplasms. An encapsulated form of PTC was first excluded, as the tumour lacked the characteristic nuclear features required for this diagnosis, including nuclear enlargement and elongation, irregular nuclear contours, chromatin clearing, nuclear grooves and pseudoinclusions [12]. Accordingly, the nuclear score was 0 according to the scoring system proposed by Nikiforov et al. [9]. Noninvasive follicular thyroid neoplasm with papillary‐like nuclear features (NIFTP) was excluded based on multiple criteria. Specifically, the absence of papillary‐like nuclear features (nuclear score = 0), the presence of unequivocal capsular and vascular invasion and the extensive papillary architecture observed in this lesion (> 50%), well above the threshold permitted for NIFTP (< 1%), collectively argue against this diagnosis [13]. Follicular tumour of uncertain malignant potential (FT‐UMP) and well‐differentiated tumour of uncertain malignant potential (WDT‐UMP) were also excluded. In both cases, the presence of clear and unequivocal capsular and vascular invasion represents an exclusion criterion. Furthermore, the degree of nuclear atypia observed in this lesion (nuclear score = 0) is insufficient to support a diagnosis of WDT‐UMP, which typically requires a nuclear score of 2–3 [9]. Finally, noninvasive encapsulated papillary *RAS*‐like thyroid tumour (NEPRAS), a recently described borderline neoplasm characterised by the absence of capsular and/or vascular invasion and by subtle (‘delicate’) nuclear changes (corresponding to a nuclear score 2 according to Nikiforov et al. [9], which is insufficient for a diagnosis of PTC [14, 15]), was excluded based on the presence of unequivocal capsular and vascular invasion and the absence of nuclear features consistent with a nuclear score of 2. Taken together, these findings support the diagnosis of an encapsulated angioinvasive follicular carcinoma showing papillary architecture, rather than a borderline or low‐risk follicular‐patterned neoplasm.

**Figure 4:**
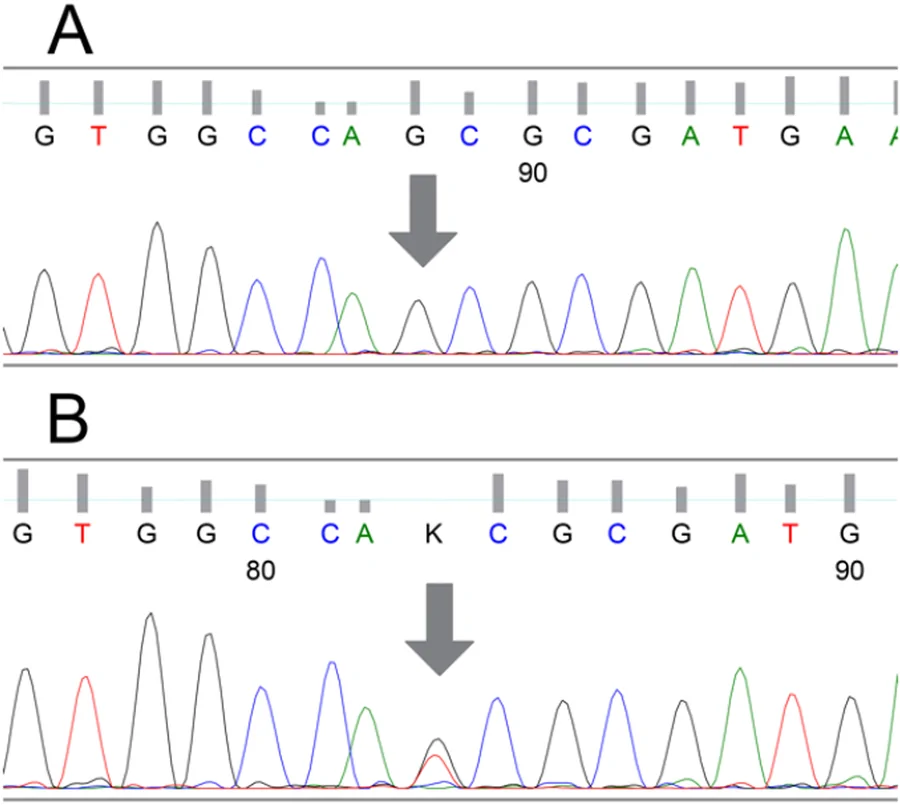
Representative Sanger sequencing electropherograms of the *GNAS* gene. Panel (A) shows the wild‐type sequence, whereas panel (B) shows the analysed sample carrying the c.681 G > T substitution. The mutated nucleotide is indicated by an arrow. The identification of a *GNAS* mutation supports the role of cAMP pathway activation in the development of autonomous function and may contribute to tumorigenesis.

Molecular testing was conducted on the primary tumour tissue. Gene selection for molecular analysis was made considering the relation of *TSH* and *GNAS* with hyperfunction in thyroid nodules [16], and of *BRAF*, *N‐RAS*, *TERT* with thyroid carcinogenesis [17, 18, 19]; finally *DICER1* has been tested for the link specifically with pediatric thyroid carcinogenesis, in particular for the relation to *DICER1*‐syndrome, predisposing to benign and malignant thyroid tumours [20]. Sequencing included *TSHR* (NM_000369.3; exons 1–10), *GNAS* (NM_000516.7; exons 8‐9), *BRAF* (NM_004333.6; exon 15), *K‐RAS* (NM_033360.2; exons 2 and 3), *H‐RAS* (NM_005343.2; exons 2 and 3), *N‐RAS* (NM_002524.3; exons 2 and 3) and *TERT promoter* (NM_198253.3), and the hotspot regions of *DICER1* (NM_177438.3; exons 24–25) by direct sequencing (SeqStudio Genetic Analyzer; Applied Biosystems, USA), as previously reported [21]. Molecular analysis performed on the histological samples identified a somatic *GNAS* mutation (c.681 G > T, p.Q227H) (Figure 4). No pathogenic variants were detected in *TSHR*, *BRAF, RAS* genes, or *TERT* promoter. Germline *DICER1* testing was negative. At 9 months of follow‐up, the patient was in clinical and biochemical remission. He was treated with levothyroxine 50 µg daily, with stable thyroid function tests (TSH 1.53 mIU/L, fT4 17 pmol/L). Serum thyroglobulin (1.33 µg/L) and TgAb (15 IU/mL) remained stable. Follow‐up neck ultrasound performed in December 2025 showed postoperative changes consistent with left hemithyroidectomy, a normal right thyroid lobe, persistence of ectopic intrathyroidal thymic tissue, and no evidence of cervical lymphadenopathy. Growth and pubertal development are currently being monitored, with mild deceleration of stature growth. As the identified *GNAS* mutation was somatic and confined to tumour tissue, no systemic or germline involvement is suspected; longitudinal auxological follow‐up is ongoing.

Written informed consent for publication of the patient's clinical details and clinical images was obtained from the patient's parents. Clinical data were retrospectively collected from medical records and fully anonymized.

### 2.1 Literature Review

To contextualise the present case, we conducted a structured narrative review of the literature on pediatric hyperfunctioning (*hot*) thyroid nodules, with the aim of evaluating the associated risk of malignancy and delineating the current gaps in knowledge. A literature search was performed using PubMed/MEDLINE, Scopus and Embase, including articles published up to September 1, 2025. The following keywords were used in various combinations: *hyperfunctioning thyroid nodule, autonomous thyroid nodule, hot nodule, thyroid carcinoma* and *pediatric* or *children*. Studies were considered eligible if they met the following criteria [1]. original case reports, case series, or clinical studies describing hyperfunctioning thyroid nodules in patients under 18 years of age [2]; histologically confirmed thyroid carcinoma arising within an autonomous nodule; and [3] availability of relevant clinical, imaging, cytological, or molecular data. Exclusion criteria included adult patients (≥ 18 years), non‐autonomous (*cold*) nodules, insufficient clinical data and non‐English publications. Given the rarity of the condition and the heterogeneity of available evidence, a formal systematic review with quantitative synthesis was not performed. Instead, relevant studies were selected and qualitatively analysed to provide a comprehensive overview of clinical, pathological and molecular features.

Reported cases were categorised into two groups: (1) clinical studies or case series (Table 1), and (2) individual case reports (Table 2).

**Table 1:** Summary of published pediatric case series of AFTNs with reported malignancy.

Study (Author, Year)	Country	Sample Size	Mean age (y)	TSH	Scintigraphy	FNAC	Histology	Malignancy rate	Notes
Hopwood et al. (1976) [22]	USA	6	11.7	Suppressed	Hot nodules		PTC	**16.6% (1/6)**	
Croom et al. (1987) [23]	USA	53		Suppressed	Hot nodules		DTC	**11.3% (6/53)**	
Niedziela et al. (2002) [24]	Poland	31	15.3	Suppressed	Hot nodules		5 PTC, 4 FTC	**29% (9/31)**	
Corrias et al. (2010) [25]	Italy	9	11.5	6 suppressed, 3 euthyroidism	Hot nodules	Indeterminate in 3/6 hyperthyroid	Hyperthyroid:1 HCA and 5 FA; Euthyroid: 1 PTC	**33.3% (1/3): euthyroid only**	
Mirfakhraee et al. (2013) [26]	Multi	77	Mixed (7.9% pediatric)	Suppressed	Hot nodules	FNAC accuracy: 43%	57% PTC, 36% FTC	**0%–12.5%**	FNAC limitations in autonomous nodules; adult and pediatric cases
Ly et al. (2016) [27]	USA	31	15	Suppressed	Hot nodules		No malignancy observed	0%	
Rosario et al. (2021) [28]	Brazil	17	14	Suppressed	Hot nodules		FTC	**5.9% (1/17)**	

Abbreviations: DTC, differentiated thyroid carcinoma; FA, follicular adenoma; FNAC, fine‐needle aspiration cytology; FTC, follicular thyroid carcinoma; HCA, Hürthle cell adenoma; PTC, papillary thyroid carcinoma; TSH, thyroid‐stimulating hormone.

**Table 2:** Published pediatric case reports of AFTNs with malignant histology.

Study (Author, Year)	Country	Age	Sex	TSH (mUI/L)	fT4 (pmol/L)	fT3 (pmol/L)	US Findings	Scintigraphy	FNAC	Surgery	Histology	Genetic
Sussman et al. (1968) [29]	USA	6 y	F	Suppressed				Hyperfunctioning area in the right lobe		TT	**PTC + FTC**	
Nagai et al. (1987) [30]	USA	11 y	F	Suppressed		Elevated	35‐mm nodule (left lobe)	Hyperfunctioning nodule		HT	**FTC with capsular invasion and angioinvasion**	
Siddiqui et al. (1995) [31]	USA	16 y	F	Normal	Slight elevated			Mildly increased uptake (left lobe)	Non diagnostic (Bethesda I)	HT → CT	**HCC**	
Cirillo et al. (1998) [32]	USA	17 y	F	< 0.03 (0.35–5.50)	Normal		21‐mm hypoechoic nodule (left lobe)	Hyperfunctioning nodule		HT + IST → CT	**PTC with lymph node metastases**	
Mircescu et al. (2000) [5]	Romania	11 y	F	0.03 (0.1–5.0)	> 75 (8.0–18.0)		40 × 22 × 20 mm, partly cystic nodule	Hyperfunctioning nodule		HT + IST → CT	**PTC**	TSHR mutation
Tfayli et al. (2010) [33]	USA	11 y	F	Suppressed	14.6 (9.4–23.7)	5.9 (1.9–3.2)	35‐mm non‐homogenous nodule (right lobe)	Predominant autonomous activity (right lobe)	Not diagnostic (Bethesda I)	HT + IST	**PTC**	
Damle et al. (2011) [34]	India	2 m	M	Suppressed	Elevated	Elevated	34 × 22 × 20 mm nodule	Hyperfunctioning nodule	Non diagnostic (Bethesda I)	HT → CT	**PTC**	
		7 y	F	0.01 (0.5–4.5)	27.9 (5.8–15.4)	7.4 (1.0–3.0)	50 × 50 mm nodule	Hyperfunctioning nodule	Benign (Bethesda II)	NTT	**FVPTC**	
Yalla et al. (2011) [35]	USA	13 y	F	0.01 (0.6–3.7)	18.0 (7.5–21.1)		35‐mm heterogeneous, hypervascular nodule (left lobe)	Hyperfunctioning nodule		HT + IST → CT	**HCC**	
Gabalec et al. (2013) [36]	Czech Republic	15 y	F	0.01 (0.3–5.6)	30.6 (7.0–15.9)		35 × 23 × 45 m cystic nodule	Hyperfunctioning nodule	Suspicious (Bethesda IV)	HT → CT	**FVPTC with vascular invasion**	
Ruggeri et al. (2013) [6]	Italy	15 y	F	0.001 (0.27–4.2)	25.9 (11.6–21.9)	7.7 (3.0–6.8)	35 × 30 × 21 mm isoechoic nodule (right lobe)	Hyperfunctioning nodule (right lobe)		Surger‐ type not specified	**FVPTC**	*TSHR* mutation and *GNAS* polymorphisms
Joshi et al. (2015) [37]	Australia	11 y	F	Suppressed			25 × 19 × 16 mm hyperechoic, hypervascular (right lobe)	Hyperfunctioning nodule (right lobe)	Suspicious (Bethesda IV)	HT → CT	**FTC angioinvasive**	
Rees et al. (2015) [38]	UK	16 y	F	< 0.03 (0.53–3.59)	39.4 (12.0–20.6)	14.3 (3.5–7.7)	40 × 25 mm hyperechoic, hypervascular nodule (left lobe)	Asymmetric uptake	Non diagnostic (Bethesda I)	HT → CT	**FVPTC**	
Blackburn et al. (2018) [7]	UK	12 y	F	< 0.03 (0.3–3.8)	10.1 (9.0–19.0)	9.1 (3.6–6.4)	21 × 17 × 17 mm heterogeneous hypervascular nodule (right lobe)			HT → CT	**FTC**	*TSHR* mutation
Dy et al. (2018) [39]	USA	14 y	F	0.02 (0.4–5.6)	18.0 (11.6–19.3)		34 × 21 × 29 mm hypoechoic, heterogenous, hypervascular nodule	Hyperfunctioning nodule	Benign (Bethesda II)	HT	**FTC with minimal capsular invasion**	
Van Vlaenderen et al. (2020) [40]	Belgium	12 y	F	< 0.005	51 (12.0–22.0)		53‐mm hypervascular solid mass with microcalcifications (left lobe)	Heterogeneous hyperfunctioning uptake (both lobes)		HT	**PTC + FTC**	

Abbreviations: CT, completion thyroidectomy (totalization); FNAC, fine‐needle aspiration cytology; fT3, free triiodothyronine; fT4, free thyroxine; FTC, follicular thyroid carcinoma; FVPTC, follicular variant of papillary thyroid carcinoma; HCC, Hürthle cell carcinoma; HT, hemithyroidectomy; IST, isthmusectomy; NTT, near‐total thyroidectomy; PTC, papillary thyroid carcinoma; TSH, thyroid‐stimulating hormone; TT, total thyroidectomy; US, ultrasound.

Individual case reports allowed us to better focus on clinical, biochemical, pathological and, when available, molecular features of these rare entities. Specifically, we focused on surgically managed hyperfunctioning nodules with histological confirmation.

Published pediatric studies described AFTNs across the entire pediatric age spectrum, from early infancy to late adolescence. Reported age at diagnosis ranged from 2 months to 17 years, with most cases occurring in school‐age children and adolescents (Tables 1 and 2). A consistent female predominance was observed across the literature, with malignant cases reported almost exclusively in female patients, as derived from individual malignant case reports (Table 2), with a median age at diagnosis of 12 years. From a biochemical standpoint, suppressed TSH levels were reported in the majority of pediatric AFTNs, reflecting autonomous thyroid hormone production. Among malignant case reports, overt biochemical hyperthyroidism was present in approximately 69% of patients (11/16), while isolated euthyroid presentations were also described, particularly in older reports [25, 31]. Clinical presentation was heterogeneous, ranging from symptomatic thyrotoxicosis to isolated cervical swelling or incidental detection, indicating that clinical features alone did not reliably predict malignant histology.

US findings in pediatric AFTNs were variable and frequently non‐specific. Nodules were typically solitary and often large. Reported nodule diameters ranged from approximately 20 mm to over 50 mm, with more than two‐thirds of malignant nodules exceeding 30 mm in maximum diameter. Most lesions were described as solid or predominantly solid, with heterogeneous echotexture and marked intra‐nodular hypervascularity. Notably, several malignant AFTNs exhibited ultrasonographic features typically considered suspicious, including irregular or multilobulated margins, cystic components and microcalcifications, despite scintigraphic evidence of autonomous function. These observations highlight that hyperfunctioning behaviour did not exclude sonographic features associated with malignancy.

FNAC demonstrated limited diagnostic performance in pediatric AFTNs. Across reported studies, cytological results frequently fell into non‐diagnostic or indeterminate categories, reflecting both sampling limitations and the intrinsic difficulty of cytological assessment in follicular‐patterned lesions. Among malignant pediatric cases, FNAC was non‐diagnostic (Bethesda I) in approximately 25% of cases (4/16) [31, 33, 34, 38], benign (Bethesda II) in 12.5% (2/16) [34, 39] and indeterminate (Bethesda III–IV) in 12.5% (2/16) [36, 37]. In several cases ultimately diagnosed as carcinoma, FNAC failed to raise preoperative suspicion. These findings were consistent with data from mixed pediatric–adult cohorts, in which FNAC accuracy in hyperfunctioning nodules was reported to be approximately 43%, with false‐negative rates reaching up to 30% [26].

Surgical management of pediatric AFTNs was heterogeneous and reflected both diagnostic uncertainty and concern regarding malignancy. Hemithyroidectomy or lobectomy represented the most frequently adopted initial surgical approach and was performed in approximately 81% of malignant cases (13/16), often based on presumed benign disease or indeterminate cytology. However, following definitive histological diagnosis, completion thyroidectomy was required in nearly 70% of these patients (9/13) [5, 7, 31, 32, 34, 35, 36, 37, 38]. Total or near‐total thyroidectomy was performed as the initial procedure in a minority of cases (12.5%, 2/16), typically in the presence of large nodules or highly suspicious imaging features [29, 34]. Overall, the literature supported a stepwise and individualised surgical strategy, with definitive oncologic management frequently guided by postoperative histology.

Reported malignancy rates in pediatric AFTNs varied widely across published studies, ranging from 0% [27] to nearly 30% [24]. This wide range likely reflected differences in study design, referral patterns, sample size and the level of histopathological detail available in individual series. While pediatric series provided estimates of overall malignancy risk, detailed histological characterization was mainly derived from individual pediatric cases, in which tumour subtype was consistently reported. Based on these cases, PTC was identified in 6 patients (37.5%) [5, 29, 32, 33, 34, 40]. Follicular‐patterned carcinomas were collectively more frequent, with FTC reported in 6 cases (37.5%) [7, 29, 30, 37, 39, 40]. and FVPTC in 4 cases (25%) [6, 34, 36, 38], together accounting for 62.5% of malignant lesions. Mixed histologies, such as synchronous PTC and FTC within the same patient, were described in isolated reports [29, 40]. Tumours historically reported as Hürthle cell carcinoma were described in older pediatric studies [31, 35]; however, the 2022 WHO classification of thyroid tumours introduced numerous changes [4], and it is therefore possible that many of these entities would now fall into different histological categories. This represents a limitation of the present review in comprehensively reclassifying these tumours according to current histopathological criteria.

Molecular analyses were reported in only a limited number of pediatric malignant AFTNs. In these cases, activating mutations of *TSHR* and *GNAS* were identified [5, 6, 7], consistent with constitutive activation of the cAMP signalling pathway underlying autonomous hormone production. In this regard, our case, the first reported in the literature of a follicular thyroid carcinoma showing papillary architecture, harbours a *GNAS* mutation (c.681 G > T, p.Q227H), which is also frequently altered in follicular adenomas showing papillary architecture [4]. This link could suggest a common molecular neoplastic pathway between follicular adenoma showing papillary architecture and the aforementioned type of thyroid carcinoma showing papillary architecture, supporting the idea of malignant transformation arising from the adenoma. Although molecular data were limited, the occurrence of these alterations in malignant tumours indicates that molecular drivers of functional autonomy may coexist with differentiated thyroid carcinoma. However, the limited number of reported cases and the absence of molecular data from benign pediatric AFTNs preclude any inference regarding a causal role of these mutations in malignant transformation or a stepwise progression from benign to malignant disease.

Overall, the available literature demonstrated that AFTNs in children cannot be uniformly considered benign. The wide variability in reported malignancy rates, limited diagnostic reliability of FNAC, frequent presence of suspicious ultrasonographic features, and occurrence of differentiated thyroid carcinomas across all pediatric age groups supported a cautious and individualised diagnostic and therapeutic approach. Within this context, the present case adds further evidence to the malignant potential of pediatric AFTNs.

## 3 Discussion

Our case expands the spectrum of pediatric malignant AFTNs, highlighting a follicular‐patterned carcinoma with extensive papillary architecture as the malignant evolution of a follicular adenoma with papillary architecture, associated with *GNAS*‐driven hyperfunction. Among the limited number of well‐documented pediatric malignant AFTNs, follicular‐patterned carcinomas (FTC and FVPTC) account for a substantial proportion of reported malignancies, contrasting with the predominance of classic PTC in nonfunctioning pediatric tumours. The predominance of follicular‐patterned lesions carries important diagnostic implications. FNAC is limited in distinguishing benign from malignant follicular neoplasms, as cytology cannot assess capsular or vascular invasion. Consequently, several malignant AFTNs, as in our case, have initially been classified as benign and only recognised as carcinomas postoperatively, underscoring the need for heightened clinical suspicion and comprehensive evaluation of large or atypical nodules, even with suppressed TSH and apparently benign scintigraphy. Importantly, this tumour does not fulfil the diagnostic criteria for papillary thyroid carcinoma, as it lacks the characteristic nuclear features of PTC. Rather, it represents a follicular‐patterned carcinoma showing papillary architecture and evidence of capsular and vascular invasion. As the risk of malignancy cannot be definitively excluded, the recent European Thyroid Association guidelines recommend surgery as the preferred treatment for the autonomous nodule in childhood, while I‐131 may be considered for small nodules (Suggestion 8A) [2].

From a molecular perspective, malignant pediatric AFTNs exhibit a genetic profile distinct from conventional pediatric thyroid carcinomas. Malignant pediatric AFTNs frequently harbour activating alterations in the *TSHR–Gsα–cAMP* pathway, in contrast to classic pediatric PTCs, which are dominated by *BRAF V600E* and *RET/PTC* rearrangements [21]. When available, molecular data indicate that approximately half of malignant pediatric AFTNs demonstrate activating *TSHR* or *GNAS* alterations, including coexistent mutations or polymorphisms, suggesting that constitutive pathway activation may drive clonal proliferation and, in some cases, secondary malignant events [5, 6, 7]. The somatic *GNAS* mutation identified in our patient aligns with these findings, emphasising the relevance of molecular profiling in guiding management. Reported malignancy rates vary due to differences in definitions of hyperfunction, scintigraphy criteria, molecular testing availability and surgical selection. Moreover, pediatric thyroid tumours also behave differently from adult thyroid cancer: children often present with larger or more advanced lesions yet achieve excellent long‐term outcomes [41, 42, 43]. These observations highlight the limitations of extrapolating adult‐based assumptions to pediatric AFTNs. Notably, no prospective, multicenter studies have systematically integrated functional, cytologic, sonographic, molecular and histopathologic data in pediatric AFTNs. This gap hinders reliable risk stratification and perpetuates reliance on adult paradigms. Even basic diagnostic tools, such as ultrasound, remain incompletely characterised in this context; features typically considered suspicious in adults (hypoechogenicity, irregular margins and microcalcifications) have uncertain predictive value in children with hyperfunctioning nodules. Accordingly, current pediatric guidelines provide limited guidance on how these findings should influence clinical management [1, 2, 44, 45].

In our case, the indication for surgery was driven by documented progressive nodule growth on serial US and the presence of subclinical hyperthyroidism, rather than by overtly suspicious cytological or ultrasonographic features. Hemithyroidectomy was chosen as the initial surgical approach in line with current pediatric practice for AFTNs [1, 2, 44, 45], enabling definitive histological diagnosis while minimising surgical morbidity. Molecular analysis was subsequently performed because of the pediatric setting and the unexpected malignant histology in a hyperfunctioning nodule, with the aim of characterising the biological basis of autonomous hormone production and excluding alternative oncogenic drivers. The identification of a somatic *GNAS* mutation supported activation of the cAMP pathway and provided molecular context to the functional phenotype observed in this pediatric tumour. Importantly, the tumour described in this case does not fulfil the diagnostic criteria for PTC, as it lacks the characteristic nuclear features of PTC. Rather, it represents a follicular‐patterned carcinoma showing papillary architecture and evidence of capsular and vascular invasion. While this morphology overlaps with that of follicular thyroid adenoma showing papillary architecture, we do not propose this lesion as a novel WHO entity. Instead, these findings support the interpretation of a phenotypic and molecular continuum between benign and malignant follicular‐patterned tumours showing papillary architecture, potentially driven by shared alterations in the cAMP signalling pathway.

Taken together, these data underscore the importance of recognising malignant hyperfunctioning thyroid nodules in children as a distinct clinicopathologic scenario, requiring careful preoperative assessment and integration of clinical, imaging and molecular findings.

From a practical standpoint, the present case and available evidence support a more nuanced approach to pediatric autonomously functioning thyroid nodules. While current guidelines generally discourage FNA in hyperfunctioning nodules and favour surgery primarily based on symptoms or size [1, 2], our findings suggest that hyperfunction alone should not be considered reassuring in children. In particular, nodules larger than 2 cm, showing progressive growth, or displaying suspicious ultrasonographic features may warrant upfront surgical management rather than observation, even in the presence of benign cytology. In this context, hemithyroidectomy represents an appropriate initial approach, allowing definitive diagnosis with limited morbidity, whereas total thyroidectomy should be reserved for cases with confirmed malignancy or high‐risk features, in line with current pediatric recommendations [1, 2, 44, 45].

## 4 Conclusions

Pediatric AFTNs cannot be considered uniformly benign, as malignancy, although uncommon, may occur, particularly in follicular‐patterned lesions that may be missed on cytology. Clinical decision‐making should therefore be guided by a comprehensive evaluation that integrates clinical presentation, ultrasonographic findings, cytology and, when available, molecular data. In this context, a cautious and individualised approach is warranted when assessing pediatric patients with hyperfunctioning nodules.

## Funding

The authors have nothing to report.

## Conflicts of Interest

The authors declare no conflicts of interest.

## Data Availability

The data supporting the findings of this study are available on request from the corresponding authors, Cristina Clausi.
